# Evaluation of Macular Thickness, Choroidal Thickness, and Choroidal Vascularity Index in Women With Polycystic Ovary Syndrome: A Cross‐Sectional Study

**DOI:** 10.1155/joph/1313399

**Published:** 2026-07-27

**Authors:** Alper Güneş, Selim Gülücü

**Affiliations:** ^1^ Department of Ophthalmology, Tokat Gaziosmanpaşa University Faculty of Medicine, Tokat, Türkiye; ^2^ Department of Obstetrics and Gynecology, Private Yenişehir Hospitals, Mersin, Türkiye

**Keywords:** choroid, choroidal vascularity index, estrogen, optical coherence tomography, polycystic ovary syndrome, polyendocrine metabolic ovarian syndrome

## Abstract

**Objective:**

To evaluate macular thickness, choroidal thickness, and choroidal vascularity index (CVI) in women with polycystic ovary syndrome (PCOS), and to assess the possible relationship between these parameters and sex hormone levels.

**Methods:**

This cross‐sectional study included 41 women with PCOS and 46 age‐matched healthy controls. All participants underwent a comprehensive ophthalmologic examination and spectral‐domain optical coherence tomography (SD‐OCT) with enhanced depth imaging (EDI) mode. The right eye of each participant was included in the analysis. Choroidal thickness was measured at five predefined locations. CVI and CVI1500 were calculated from binarized EDI‐OCT images using ImageJ software. Hormonal, metabolic, and lipid parameters were recorded. Statistical analyses included independent samples *t*‐tests and multivariable linear regression models.

**Results:**

Subfoveal and 1000 µm nasal choroidal thicknesses were significantly greater in the PCOS group (*p* = 0.048 and 0.035, respectively). CVI and CVI1500 values were significantly higher in PCOS patients (*p* = 0.027 and < 0.001, respectively), with CVI1500 demonstrating the largest effect size (Cohen’s *d* = 1.00). LA1500 was significantly higher in the PCOS group (0.108 ± 0.016 vs. 0.101 ± 0.013, *p* = 0.022), whereas SA1500 and macular thickness did not differ significantly between groups. No significant difference was observed in retinal nerve fiber layer thickness. In the systemic profile, BMI, fasting glucose, triglycerides, total cholesterol, LDL cholesterol, TSH, and T4 were comparable between groups. PCOS patients had significantly higher LH, LH/FSH ratio, total testosterone, DHEA‐SO4, insulin, HOMA‐IR levels, and lower HDL cholesterol levels. FSH was borderline lower in the PCOS group but did not reach statistical significance.

**Conclusions:**

PCOS is associated with increased choroidal vascularity and selected choroidal thickness parameters. CVI may represent a potential imaging marker of subclinical choroidal vascular alterations in PCOS.

## 1. Introduction

Polycystic ovary syndrome (PCOS) is a common endocrine disorder affecting women of reproductive age, with a global prevalence of 8%–13%, characterized by oligo/anovulation, hyperandrogenism, and polycystic ovarian morphology [1]. Through a recent international consensus process, the condition has been renamed polyendocrine metabolic ovarian syndrome (PMOS) to better reflect its multisystem endocrine, metabolic, and ovarian features and to avoid the misleading implication of pathological ovarian cysts [2]. Because existing diagnostic criteria, prior ophthalmic literature, and indexing systems still use PCOS, and given the recommended 3‐year transition period, the term PCOS is retained throughout the present manuscript. While its systemic and metabolic effects are well documented, its impact on ocular structures has received less attention in clinical research.

Steroid hormone receptors, including estrogen and androgen receptors, have been identified in several ocular tissues such as the conjunctiva, cornea, meibomian glands, retina, and choroid [3–5]. These findings suggest that hormonal fluctuations in PCOS may influence ocular anatomy and physiology, potentially including hormonally responsive and vascular structures such as the macula and choroid [6].

Spectral‐domain optical coherence tomography (SD‐OCT) with enhanced depth imaging (EDI) mode enables detailed evaluation of the choroid and retina. Previous studies have demonstrated alterations in macular and choroidal thickness (ChT) in PCOS patients [7, 8]. Recently, the choroidal vascularity index (CVI) has been introduced as a quantitative marker of the vascular component of the choroid. The CVI is defined as the ratio of luminal area (LA) to total choroidal area (TCA) and may provide additional insight beyond thickness measurements alone, as it is considered more stable and less susceptible to physiological variation [9].

Given that the choroid is primarily responsible for metabolic support of the outer retina, vascular changes in this layer may reflect systemic hormonal effects in PCOS [10]. However, data on CVI in PCOS remain scarce. This study aimed to evaluate macular thickness, ChT, and CVI in women with PCOS and to investigate their possible association with circulating sex hormone levels.

## 2. Materials and Methods

This cross‐sectional study was conducted between August and September 2023 at the Departments of Ophthalmology and Obstetrics and Gynecology, Tokat Gaziosmanpaşa University Faculty of Medicine, Türkiye. The study adhered to the Declaration of Helsinki and was approved by the local clinical research ethics committee (Approval No: 20‐KAEK‐268). Written informed consent was obtained from all participants. A preliminary version of this manuscript was previously deposited as a preprint [11].

### 2.1. Study Population

A total of 41 women with PCOS and 46 age‐matched healthy controls were included. PCOS diagnosis was based on the Rotterdam 2003 criteria, which require at least two of the following: oligo/anovulation, clinical or biochemical hyperandrogenism, and polycystic ovarian morphology on ultrasound [1]. Subjects with other endocrine disorders (e.g., pituitary insufficiency, hyperprolactinemia, and congenital adrenal hyperplasia), systemic diseases (e.g., diabetes mellitus and hypertension), or ocular pathologies were excluded. Additional exclusion criteria were a spherical equivalent > ± 2.0 D, axial length < 20 mm or > 26 mm, history of ocular trauma or surgery, smoking, pregnancy, current use of oral contraceptives or other hormonal therapy, use of medications known to affect vascular function (e.g., antihypertensives, vasodilators), or current use of medications unrelated to PCOS.

### 2.2. Ophthalmologic Examination and Imaging

All participants underwent a standardized ophthalmologic examination including best‐corrected visual acuity (BCVA) using a Snellen chart, intraocular pressure (IOP) measurement via applanation tonometry, slit‐lamp biomicroscopy, dilated fundus examination, and SD‐OCT imaging. Examinations were conducted between 09:00 and 12:00 to minimize diurnal variation [12]. The right eye of each participant was included in the analysis.

OCT scans were performed using a Cirrus 5000 SD‐OCT device (Carl Zeiss Meditec, Dublin, CA, USA) in EDI mode. ChT was measured manually as the vertical distance from the outer border of the hyperreflective retinal pigment epithelium (RPE) to the inner scleral boundary. All ChT and CVI measurements were performed independently by two trained observers from the Department of Ophthalmology. OCT images were analyzed before merging with the gynecologic and hormonal datasets, thereby minimizing observer bias. Each image was measured independently by both observers, and the mean of the two measurements was used for the final statistical analyses. Interobserver reproducibility was assessed using intraclass correlation coefficients using a two‐way random‐effects model with absolute agreement. ICC values ranged from 0.88 to 0.92 across ChT and choroidal vascular parameters, indicating good‐to‐excellent interobserver agreement according to the Koo and Li classification. Detailed ICC values for individual parameters are presented in Table 1. Measurements were obtained at five locations: subfoveal, 1000 µm and 2000 µm nasal, and 1000 µm and 2000 µm temporal to the fovea [13].

**TABLE 1:** Interobserver reliability of choroidal and macular measurements.

Parameter	ICC	Interpretation
TCA	0.89	Good
LA	0.89	Good
SA	0.88	Good
CVI	0.89	Good
TCA1500	0.91	Excellent
LA1500	0.90	Good
SA1500	0.91	Excellent
CVI1500	0.91	Excellent
Subfoveal choroidal thickness	0.91	Excellent
Choroidal thickness, 1000 µm nasal	0.90	Excellent
Choroidal thickness, 2000 µm nasal	0.88	Good
Choroidal thickness, 1000 µm temporal	0.92	Excellent
Choroidal thickness, 2000 µm temporal	0.89	Good

*Note:* ICC values were calculated using a two‐way random‐effects model with absolute agreement, based on paired Set 1 and Set 2 measurements. Interpretation per Koo and Li (2016): < 0.50 = poor; 0.50–0.75 = moderate; 0.75–0.90 = good; > 0.90 = excellent.

Abbreviations: CVI, choroidal vascularity index; LA, luminal area; SA, stromal area; TCA, total choroidal area.

### 2.3. CVI Measurement

**FIGURE 1:**
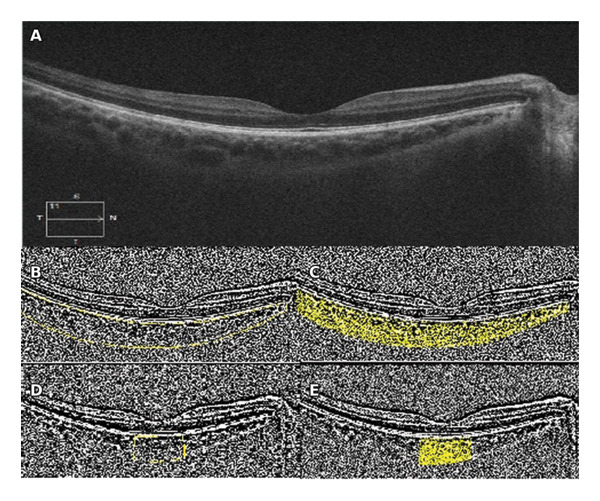
Representative enhanced depth imaging optical coherence tomography images used for choroidal vascularity index analysis. (A) Original EDI‐OCT image. (B) Choroidal area manually delineated between the outer border of the retinal pigment epithelium and the choroid–scleral interface. (C) Image binarized using the Niblack auto‐local thresholding method. (D) Luminal and stromal areas identified after binarization. (E) Regional 1500 µm fovea‐centered area used for CVI1500 analysis.

The CVI was calculated using ImageJ software (Version 1.53i, NIH, USA) based on the method described by Agrawal et al. [9]. EDI‐OCT images were binarized using Niblack’s auto‐local thresholding. The TCA was outlined using the polygon tool. LA was defined as the dark pixels, and stromal area (SA) as the light pixels. CVI was computed as the ratio of LA to TCA. Regional analysis was also performed in a circular area of 1500 µm centered on the fovea (CVI1500). TCA, LA, and SA were additionally calculated within a 1500‐µm fovea‐centered circular region and were denoted as TCA1500, LA1500, and SA1500, respectively. (see Figure 1).

### 2.4. Hormonal Assessment

Peripheral venous blood samples were collected from all participants between Days 2 and 4 of the menstrual cycle. Serum hormonal measurements, including follicle‐stimulating hormone (FSH), luteinizing hormone (LH), estradiol (E2), prolactin, total testosterone, dehydroepiandrosterone sulfate (DHEA‐SO4), insulin, thyroid‐stimulating hormone (TSH), and thyroxine (T4), were performed using a Roche Cobas e601 analyzer (Roche Diagnostics GmbH, Germany) based on the electrochemiluminescence immunoassay (ECLIA) method. Fasting blood glucose levels were also measured, and the homeostatic model assessment for insulin resistance (HOMA‐IR) was calculated as fasting blood glucose (mg/dL) × fasting insulin (µIU/mL)/405. The LH/FSH ratio was calculated for each participant and included in the statistical analyses. A fasting lipid panel comprising triglycerides, total cholesterol, low‐density lipoprotein (LDL) cholesterol, and high‐density lipoprotein (HDL) cholesterol was measured on a Roche Cobas *c*‐series chemistry analyzer using standard enzymatic colorimetric methods.

### 2.5. Statistical Analysis

Statistical analyses were performed using SPSS Version 22.0 (IBM Corp., Armonk, NY, USA). Normality was assessed using Kolmogorov–Smirnov and Shapiro–Wilk tests. Data were expressed as mean ± standard deviation (SD). Independent samples *t*‐test was used to compare variables between groups. A multivariable linear regression analysis was performed to evaluate the association between subfoveal ChT and hormonal parameters. In addition to LH/FSH ratio, estradiol, prolactin, total testosterone, and insulin, age, body mass index (BMI), axial length, and spherical equivalent were included in the adjusted model as potential confounding variables. Between‐group effect sizes were quantified using Cohen’s d for the principal continuous outcomes. Ninety‐five percent confidence intervals (CIs) for the between‐group mean differences were also calculated for the principal choroidal parameters. A *p* value < 0.05 was considered statistically significant.

## 3. Results

A total of 87 eyes (41 PCOS, 46 controls) were analyzed. There were no statistically significant differences between groups in age, BMI, axial length, IOP, or spherical equivalent (Table 2). Height was marginally lower in the PCOS group (*p* = 0.048). PCOS patients had significantly higher LH, LH/FSH ratio, total testosterone, DHEA‐SO4, insulin, and HOMA‐IR levels, while HDL cholesterol was significantly lower. FSH was borderline lower in the PCOS group but did not reach statistical significance (*p* = 0.055). Estradiol, prolactin, TSH, T4, fasting glucose, triglycerides, total cholesterol, and LDL cholesterol were comparable between groups. All participants had a BCVA of 20/20, corresponding to 0.0 logMAR.

**TABLE 2:** Demographic, clinical, hormonal, metabolic, thyroid, and lipid characteristics of PCOS patients and healthy controls.

Characteristic	PCOS (*n* = 41), mean ± SD	Control (*n* = 46), mean ± SD	*p* value
Age (years)	22.49 ± 1.58	22.63 ± 1.64	0.681
Height (cm)	162.85 ± 5.53	165.26 ± 5.62	**0.048** ^**∗∗**^
Body weight (kg)	58.02 ± 10.09	58.83 ± 9.88	0.709
BMI (kg/m^2^)	21.83 ± 3.33	21.54 ± 3.47	0.692
Axial length (mm)	22.4 ± 0.3	22.8 ± 0.6	0.715
Intraocular pressure (mmHg)	15.1 ± 2.4	14.8 ± 2.3	0.532
Spherical equivalent (D)	−0.19 ± 0.48	−0.16 ± 0.61	0.672
FSH (mIU/mL)	5.46 ± 0.96	5.87 ± 1.00	0.055
LH (mIU/mL)	9.88 ± 6.47	5.49 ± 2.02	**0.001** ^**∗∗**^
LH/FSH ratio	1.72 ± 1.31	0.94 ± 0.33	**< 0.001** ^**∗∗**^
Estradiol (pg/mL)	47.13 ± 30.54	40.05 ± 12.59	0.153
Total testosterone (ng/mL)	0.43 ± 0.19	0.31 ± 0.21	**0.007** ^**∗∗**^
Prolactin (ng/mL)	21.43 ± 9.30	18.97 ± 8.82	0.210
DHEA‐SO4 (µg/dL)	316.81 ± 118.98	262.37 ± 62.77	**0.011** ^**∗∗**^
Insulin (µIU/mL)	20.29 ± 3.84	11.93 ± 10.79	**0.001** ^**∗∗**^
HOMA‐IR	4.51 ± 0.96	2.70 ± 2.66	**0.001** ^**∗∗**^
TSH (µIU/L)	1.60 ± 0.68	1.80 ± 0.96	0.272
T4 (µg/dL)	1.27 ± 0.19	1.28 ± 0.16	0.817
Fasting glucose (mg/dL)	89.87 ± 7.26	89.77 ± 6.18	0.946
Triglycerides (mg/dL)	81.24 ± 29.84	74.60 ± 30.45	0.307
Total cholesterol (mg/dL)	157.05 ± 20.92	158.67 ± 29.02	0.764
HDL cholesterol (mg/dL)	49.66 ± 9.25	61.33 ± 13.82	**0.001** ^**∗∗**^
LDL cholesterol (mg/dL)	91.15 ± 19.25	91.83 ± 23.62	0.884

*Note:* DHEA‐SO4, dehydroepiandrosterone sulfate; HOMA‐IR, homeostatic model assessment for insulin resistance, T4: thyroxine. Bold values indicate statistically significant between‐group differences at *p* < 0.05.

Abbreviations: HDL, high‐density lipoprotein; LDL, low‐density lipoprotein; TSH, thyroid‐stimulating hormone.

^∗∗^ *p* < 0.05.

ChT was numerically greater in the PCOS group at all five measurement points. Statistically significant increases were observed at the subfoveal location (*p* = 0.048; mean difference 28.7 µm, 95% CI 0.3 to 57.1) and at 1000 µm nasal to the fovea (*p* = 0.035; mean difference 29.8 µm, 95% CI 2.7 to 56.9). The 1000‐µm temporal location demonstrated a numerical increase with a comparable effect size (Cohen’s *d* = 0.41) but did not reach the conventional threshold of statistical significance (*p* = 0.067). No significant differences were observed at the 2000‐µm nasal or temporal points (*p* = 0.448 and 0.284, respectively).

Quantitative analysis of binarized OCT images revealed significantly higher LA (*p* = 0.018; mean difference 0.080, 95% CI 0.022 to 0.138), TCA1500 (*p* = 0.001; mean difference 0.030, 95% CI 0.015 to 0.045), and LA1500 in PCOS patients. LA1500 measurement averages were significantly higher in the PCOS group than in healthy controls (0.108 ± 0.016 vs. 0.101 ± 0.013, *p* = 0.022; mean difference 0.007, 95% CI 0.001 to 0.013). TCA was numerically greater in the PCOS group with a comparable effect size (Cohen’s *d* = 0.48) but did not reach the conventional significance threshold (*p* = 0.061). Although SA1500 was higher in the PCOS group, this difference was not statistically significant (0.066 ± 0.010 vs. 0.062 ± 0.009, *p* = 0.118). CVI and CVI1500 were significantly elevated in the PCOS group (*p* = 0.027 and < 0.001, respectively; mean difference 0.020 [95% CI 0.003 to 0.037] and 0.020 [95% CI 0.011 to 0.029], respectively), with CVI1500 demonstrating the largest between‐group difference. Macular thickness was lower in the PCOS group; however, the difference was not statistically significant (224.6 ± 40.5 µm vs. 236.3 ± 35.7 µm, *p* = 0.181). No significant difference was observed in retinal nerve fiber layer thickness (RNFL, *p* = 0.548; Table 3).

**TABLE 3:** Comparison of choroidal parameters and macular thickness between PCOS and control groups.

Variables	PCOS (mean ± SD)	Control (mean ± SD)	*p* value
TCA (mm^2^)	0.68 ± 0.15	0.61 ± 0.14	0.061
LA (mm^2^)	0.49 ± 0.14	0.41 ± 0.13	0.018^∗∗^
SA (mm^2^)	0.23 ± 0.06	0.22 ± 0.06	0.392
CVI	0.65 ± 0.04	0.63 ± 0.04	0.027^∗∗^
TCA1500 (mm^2^)	0.18 ± 0.04	0.15 ± 0.03	0.001^∗∗^
LA1500 (mm^2^)	0.108 ± 0.016	0.101 ± 0.013	0.022^∗∗^
SA1500 (mm^2^)	0.066 ± 0.010	0.062 ± 0.009	0.118
CVI1500	0.66 ± 0.02	0.64 ± 0.02	< 0.001^∗∗^
Subfoveal ChT (µm)	353.8 ± 66.0	325.1 ± 66.8	0.048^∗∗^
ChT 1000 µm nasal (µm)	351.0 ± 66.4	321.2 ± 60.9	0.035^∗∗^
ChT 2000 µm nasal (µm)	327.1 ± 56.2	317.8 ± 55.7	0.448
ChT 1000 µm temporal (µm)	357.8 ± 55.4	334.6 ± 58.5	0.067
ChT 2000 µm temporal (µm)	317.9 ± 48.6	305.4 ± 56.2	0.284
RNFL (µm)	103.1 ± 8.1	104.0 ± 7.0	0.548
Macular thickness (µm)	224.6 ± 40.5	236.3 ± 35.7	0.181

*Note:* CVI and CVI1500 values were computed on a per‐subject basis (LA/TCA for each eye) and then averaged across the group; the group‐mean CVI is therefore not algebraically equivalent to the ratio of group‐mean LA to group‐mean TCA; ChT, choroidal thickness.

Abbreviations: CVI, choroidal vascularity index; LA, luminal area; RNFL, retinal nerve fiber layer; SA, stromal area; TCA, total choroidal area.

^∗∗^ *p* < 0.05.

Among all measured parameters, CVI1500 demonstrated the largest effect size (Cohen’s *d* = 1.00), indicating a large‐magnitude between‐group difference (large effect size). TCA1500 also showed a large effect size (*d* = 0.86), while LA and CVI demonstrated medium effect sizes (*d* = 0.59 and 0.50, respectively). Subfoveal ChT and 1000 µm nasal ChT showed small‐to‐medium effect sizes (*d* = 0.43 and 0.47, respectively).

In the adjusted multivariable regression model, both estradiol and the LH/FSH ratio showed positive associations with subfoveal ChT in the PCOS group. Estradiol demonstrated a borderline association (*β* = 0.353, *p* = 0.050), while the LH/FSH ratio showed a positive trend close to the conventional threshold for statistical significance (*β* = 0.342, *p* = 0.053). Although these findings do not allow a definitive conclusion regarding independent hormonal predictors, the similar direction and magnitude of the standardized coefficients suggest that estrogenic activity and gonadotropin balance may both contribute to ChT variation in PCOS. These regression results should therefore be considered exploratory. (see Table 4).

**TABLE 4:** Adjusted multivariable linear regression analysis of subfoveal choroidal thickness on hormonal parameters.

Variables	PCOS *β*	PCOS *p*	Control *β*	Control *p*
LH/FSH	0.342	0.053	0.318	0.039
Estradiol	0.353	0.050	0.096	0.124
Prolactin	−0.375	0.569	−0.352	0.353
Testosterone	−0.138	0.108	−0.246	0.119
Insulin	0.075	0.264	0.107	0.297

*Note:* Dependent variable: subfoveal choroidal thickness. Models were adjusted for age, body mass index, axial length, and spherical equivalent. These covariates are not shown in the table for brevity. *β* values represent standardized regression coefficients. Full regression output is available from the corresponding author upon reasonable request.

## 4. Discussion

PCOS, recently renamed PMOS to reflect its multisystem nature [2], is a prevalent endocrine disorder with systemic consequences that extend well beyond reproductive health. Affected women frequently exhibit metabolic comorbidities such as insulin resistance, obesity, dyslipidemia, and cardiovascular disease [14]. Importantly, recent evidence has demonstrated the presence of sex steroid hormone receptors in ocular tissues, providing a biological rationale for hormonally mediated effects on ocular structure and function [15].

In the present cohort, ChT was greater in PCOS patients at all five measurement points, reaching statistical significance at the subfoveal (*p* = 0.048) and 1000 µm nasal (*p* = 0.035) locations. The 1000‐µm temporal location showed a comparable directional increase that did not cross the conventional significance threshold (*p* = 0.067). These observations are broadly consistent with those of Açmaz et al., who reported increased ChT in PCOS and attributed the difference to vasodilatory effects of unopposed estrogen [13]. Estrogen receptor‐mediated modulation of vascular tone has also been demonstrated in systemic vascular studies, supporting a biologically plausible, though indirect, mechanism [16]. In contrast, Sogawa et al. found no significant relationship between choroidal blood flow and thickness in healthy individuals [17], a discrepancy that may reflect the differing systemic hormonal backgrounds of unaffected subjects compared with women with PCOS.

Beyond conventional thickness measurements, the most robust and consistent alterations in our cohort were observed in choroidal vascular parameters, particularly CVI and CVI1500. The elevation of CVI1500 (Cohen’s *d* = 1.00), which reflects a higher per‐subject luminal‐to‐TCA ratio at the macular center in PCOS eyes, suggests a relative expansion of the vascular luminal compartment. Although SA at the macular center (SA1500) was numerically higher in PCOS patients, this difference did not reach statistical significance. Because TCA1500 was also significantly greater in the PCOS group, these findings are best interpreted as regional choroidal vascular remodeling with a luminal predominance rather than as isolated luminal enlargement. Overall, our findings contribute to the growing literature on choroidal vascular changes in PCOS and highlight the CVI as a more sensitive marker than thickness measurements.

In the adjusted multivariable regression model, both estradiol and the LH/FSH ratio showed positive associations with subfoveal ChT in the PCOS group. Estradiol demonstrated a borderline association (*β* = 0.353, *p* = 0.050), while the LH/FSH ratio showed a positive trend close to the conventional threshold for statistical significance (*β* = 0.342, *p* = 0.053). The LH/FSH ratio also reached a statistically significant association in the control group, further supporting the possibility that gonadotropin balance may be linked to subclinical choroidal vascular variation. Although these findings do not permit a definitive conclusion regarding independent hormonal predictors, the similar direction and magnitude of the standardized coefficients suggest that estrogenic activity and gonadotropin balance may both contribute to ChT variation in PCOS. Given the borderline *p* values, these associations should be treated with caution and verified in larger, prospective studies.

With respect to the inner retina, OCT angiography studies such as the one by Yener et al. did not reveal differences in macular or optic disc capillary density between PCOS and controls, although increased parafoveal thickness was reported [18]. In the present study, RNFL was comparable between groups, and macular thickness was numerically lower in the PCOS group but did not reach statistical significance (224.6 ± 40.5 µm vs. 236.3 ± 35.7 µm, *p* = 0.181). The discrepancy with the parafoveal thickening reported by Yener et al. may reflect differences in measurement protocols (central macular versus parafoveal regions), study populations, or disease duration. Because visual function was preserved in our cohort, this finding should be interpreted as a non‐significant structural trend rather than as evidence of clinically relevant retinal thinning. De Souza‐Júnior et al. reported increased macular thickness in PCOS patients with insulin resistance, possibly due to Müller cell hypertrophy and associated intracellular edema [19]. Our findings did not demonstrate a statistically significant between‐group difference in macular thickness despite higher insulin and HOMA‐IR values and lower HDL cholesterol levels in the PCOS group. Differences in OCT segmentation protocols, retinal subfield definitions, disease phenotype, disease duration, or the degree and chronicity of metabolic disturbance may partly account for the variability across studies. The macular thickness findings in PCOS should therefore be interpreted cautiously and confirmed in longitudinal studies. The clinical expression of PCOS may also vary across the reproductive lifespan, and differences in disease duration or disease stage may contribute to variability in the ocular findings across studies [20].

Our findings should also be considered alongside other recent studies of the choroid in PCOS. A recent study by Icoz et al. (2026) evaluated choroidal vascular parameters in newly diagnosed PCOS patients and reported no significant differences in CVI or ChT between groups [21]. This discrepancy may be explained by several factors, including differences in disease duration, hormonal milieu, sample characteristics, and image analysis protocols. In our cohort, disease duration was not systematically recorded, which precludes formal stage‐based comparison and is acknowledged as a limitation of the present study.

A subsequent systematic review and meta‐analysis by Bousamri et al. synthesizing OCT and OCTA findings from 15 studies including 1,370 women with PCOS further contextualizes this heterogeneity [22]. Although pooled analysis demonstrated increased subfoveal ChT in PCOS overall (MD = +58.03 µm), heterogeneity was extremely high (*I* ^2^ = 99%), and the certainty of evidence was rated as very low according to the GRADE framework. Subgroup analysis showed that significant choroidal thickening was confined to studies in which disease duration was unspecified, whereas newly diagnosed cohorts demonstrated no statistically significant difference. These observations support the concept that choroidal remodeling in PCOS may evolve progressively over time rather than being present at initial diagnosis. Our preprint was included in this meta‐analysis, which means our findings should be viewed as one of several heterogeneous studies on this topic rather than as an independent confirmation of the pooled estimates.

From a translational perspective, the CVI may represent a more stable and potentially clinically relevant marker of choroidal vascular status than ChT alone, as it is less affected by physiologic variables such as age, refractive status, and diurnal variation. Alterations in the CVI may therefore provide additional insight into systemic vascular changes associated with PCOS, although their relevance to long‐term cardiometabolic risk remains to be determined.

From a clinical standpoint, the elevation of CVI observed in our PCOS cohort may reflect early subclinical microvascular alteration rather than overt structural choroidal damage, particularly given that macular thickness and RNFL were preserved in this relatively young population. As a noninvasive imaging readout, the CVI may also have a potential value as a surrogate marker of systemic vascular risk in PCOS, a population in which long‐term cardiovascular morbidity is increased and accessible vascular biomarkers are limited. Whether early CVI alterations precede or predict subsequent ocular or systemic vascular pathology cannot be addressed by the present cross‐sectional design and would require prospective, longitudinal investigation, ideally combining structural CVI with OCTA‐based microvascular metrics, and cardiovascular surrogates such as carotid intima‐media thickness or endothelial function.

### 4.1. Limitations

Several limitations of this study should be acknowledged. First, the cross‐sectional design precludes causal inference between hormonal parameters and choroidal changes. Second, the study was conducted at a single center with a relatively small sample size, which may have limited statistical power for detecting small effect sizes; this likely contributed to the nonsignificant directional differences observed for several choroidal parameters (TCA, SA1500, and 1000 µm temporal ChT) despite consistent effect directions and small‐to‐medium effect sizes. Third, the cohort consisted exclusively of young Turkish women, which may limit generalizability to other ethnic groups and age ranges. Fourth, PCOS phenotypes were not subclassified, and the hormonal heterogeneity inherent to PCOS may have influenced the observed associations. Fifth, disease duration was not systematically recorded, which precludes formal stage‐based analysis of choroidal involvement. Sixth, blood pressure measurements were not systematically recorded, which precluded formal assessment of this hemodynamic and vascular confounder. Seventh, despite age, BMI, axial length, IOP, and spherical equivalent being comparable between groups, mean height differed marginally between the PCOS and control groups (162.85 ± 5.53 cm vs. 165.26 ± 5.62 cm, *p* = 0.048); this difference is small (∼2.4 cm) and unlikely to be of biological relevance for choroidal measurements, especially given matched axial length and refractive status, but is acknowledged for completeness. Eighth, OCT angiography was not available on the imaging platform used in this protocol; the absence of OCTA‐based microvascular density and flow data restricts our ability to corroborate structural CVI findings at the capillary level, and future studies combining CVI with OCTA microvascular metrics are recommended.

## 5. Conclusion

In this cross‐sectional study, women with PCOS demonstrated increased choroidal vascularity, with CVI and CVI1500 showing the largest and most consistent between‐group differences, accompanied by greater subfoveal and 1000 µm nasal ChT. Other ChT parameters exhibited directional but nonsignificant trends, whereas macular thickness and RNFL did not differ between groups. In adjusted regression analyses, estradiol and the LH/FSH ratio showed borderline positive associations with subfoveal ChT; these preliminary findings need to be confirmed in larger studies.

These findings should be interpreted in the context of a heterogeneous literature, in which disease duration, hormonal status, PCOS phenotype, and methodological differences appear to influence the reported choroidal changes. Among the parameters we evaluated, the CVI seems to be a more stable and clinically useful marker of choroidal vascular status than thickness alone because it is less affected by physiological factors such as age, refractive error, and diurnal variation. Whether the CVI changes observed in PCOS represent early subclinical vascular remodeling with long‐term cardiometabolic implications is still unclear. Larger, longitudinal, and phenotype‐stratified studies are needed to confirm these findings. Combining CVI with OCT angiography‐based microvascular metrics in such studies may also help clarify the time course and clinical relevance of choroidal changes in PCOS.

## Author Contributions

Alper Güneş contributed to the conception and design of the study, data collection, ophthalmological examinations, OCT and CVI analyses, interpretation of the results, manuscript drafting, and critical revision of the manuscript. Selim Gülücü contributed to patient recruitment, clinical evaluation of the PCOS group, data interpretation, and critical revision of the manuscript.

## Funding

This research received no specific grant from any funding agency in the public, commercial, or not‐for‐profit sectors.

## Disclosure

Both authors read and approved the final version of the manuscript.

## Conflicts of Interest

The authors declare no conflicts of interest.

## Data Availability

The data that support the findings of this study are available from the corresponding author upon reasonable request. The data are not publicly available due to privacy or ethical restrictions.
